# Predictive value of coagulation profiles for Kawasaki disease shock syndrome: a prospective cohort study

**DOI:** 10.3389/fped.2024.1450710

**Published:** 2024-08-16

**Authors:** Bowen Li, Xiaoliang Liu, Shuran Shao, Ping Wu, Mei Wu, Lei Liu, Yimin Hua, Hongyu Duan, Kaiyu Zhou, Chuan Wang

**Affiliations:** ^1^ Department of Pediatric Cardiology, West China Second University Hospital, Sichuan University, Chengdu, Sichuan, China; ^2^ The Cardiac Development and Early Intervention Unit, West China Institute of Women and Children’s Health, West China Second University Hospital, Sichuan University, Chengdu, Sichuan, China; ^3^ West China Clinical Medical College of Sichuan University, Chengdu, Sichuan, China; ^4^ Key Laboratory of Birth Defects and Related Diseases of Women and Children (Sichuan University), Ministry of Education, Chengdu, Sichuan, China; ^5^ Key Laboratory of Development and Diseases of Women and Children of Sichuan Province, West China Second University Hospital, Sichuan University, Chengdu, Sichuan, China

**Keywords:** Kawasaki disease, coronary artery lesions, antithrombin III, D-dimer, coagulation profile

## Abstract

**Background:**

Kawasaki disease (KD) is characterized as an acute febrile inflammatory disorder, which may potentially escalate into a more severe condition termed Kawasaki disease shock syndrome (KDSS). The objective of this research is to understand the clinical attributes of KDSS and to explore the predictive significance of coagulation profiles in the incidence of KDSS.

**Method:**

Patients with Kawasaki disease (KD) were prospectively enrolled and divided into the KDSS group (*n* = 29) and the non-KDSS group (*n* = 494). Multivariate logistic regression analysis was used to ascertain the relationship between coagulation profiles and KDSS. Furthermore, ROC curve analysis was conducted to evaluate the predictive value of the coagulation profile for the occurrence of KDSS.

**Result:**

Among the KDSS patients, the median age was higher and cervical lymph node involvement was greater compared to the non-KDSS group. Additionally pericardial effusion, valve regurgitation, cardiac enlargement, coronary artery lesions (CALs), and Intravenous immunoglobulin (IVIG) resistance were significantly more frequent in the KDSS group than in non-KDSS group. Notably, Prothrombin time (PT), activated partial thromboplastin time (APTT), D-dimer, and fibrin degradation products (FDP) were significantly elevated in the KDSS group compared to the non-KDSS group. Conversely, total thrombin time (TT), fibrinogen, and antithrombin III (ATIII) activity were significantly reduced. Multivariate logistic regression analysis revealed that PT, APTT, D-dimer, and ATIII were independent risk factors for predicting KDSS occurrence. ROC curve analysis established critical values for PT, D-dimer, FDP, and ATIII as 13.45 s, 2.03 mg/L, 7.45 μg/ml, and 77.5%, respectively. Sensitivity for predicting KDSS occurrence was 76%, 79%, 83%, and 76%, while specificity was 51%, 72%, 63%, and 80%, respectively. When we performed a combined ROC curve analysis of the four indicators, we found that its predictive sensitivity was much higher. Moreover, the Delong test results showed that the AUC of the combined analysis was significantly higher than that of the individual analyses.

**Conclusion:**

Characteristic features of KDSS include older age, a greater likelihood of experiencing pericardial effusion, valve regurgitation, cardiac enlargement, CALs, and IVIG resistance. KD patients with a hypercoagulable state during the acute phase are at a higher risk of developing KDSS.

## Introduction

Kawasaki Disease (KD) is an acute, self-limiting febrile illness of unknown etiology, primarily affecting children under the age of five. It is currently the most common cause of acquired heart disease in children in developed countries (1). In recent years, some KD patients have been reported to experience hemodynamic instability during the acute phase of the disease. Sporadic cases of KD with shock syndrome were documented in the mid-1990s (2–5). Kanegaye et al. further defined Kawasaki Disease Shock Syndrome (KDSS) in 2009 (6), noting that it was associated with more severe laboratory inflammatory markers, larger coronary artery lesions (CALs), mitral valve regurgitation, and an increased risk of prolonged myocardial dysfunction (6–10). Approximately 7% of KD patients develop KDSS (6). Clinical features of KDSS include poor perfusion or shock-like states, which may present early with atypical KD symptoms, making it challenging to diagnose (11). Recent studies have also highlighted difficulties in early recognition, delayed treatment, and an increased need for IVIG retreatment in KDSS cases in North America and Taiwan (5–7, 9, 12). Therefore, early identification and treatment of KDSS patients are crucial to reducing systemic and vascular inflammation.

However, research on predicting KDSS is relatively limited. A case-control study found that KD patients with IL-6 levels higher than 66.7 pg/ml, IL-10 levels higher than 20.85 pg/ml, and IFN-γ levels higher than 8.35 pg/ml are at a higher risk of developing KDSS (13). However, these findings were limited by a small sample size and costly indexes. Moreover, previous studies have summarized the clinical characteristics of KDSS but have not developed a useful predictive model for KDSS. Therefore, an effective and inexpensive predictive indicator is needed to guide clinical work for early KDSS prediction.

It is well-known that acute inflammatory reactions can upregulate cytokine expression and activate the coagulation system (14). In a study of critically ill patients, Ogura et al. found that coagulation abnormalities were associated with an increase in the Acute Physiology and Chronic Health Evaluation (APACHE) II score, reflecting systemic inflammatory response syndrome (15). Furthermore, the relationship between immune system activation and the coagulation system is evident in systemic autoimmune or immune-mediated diseases. Encouragingly, our previous studies, along with others, have found that the balance between the coagulation and fibrinolysis systems is disrupted during the acute phase of KD (16–22). Consequently, we speculate that this condition may be more severe in patients with KDSS, and these coagulation-related indicators could serve as predictors for KDSS in clinical settings.

Therefore, in the present study, we prospectively investigated the coagulation profiles in patients with KDSS to test the hypothesis that coagulation-related indicators are valuable for predicting KDSS for the first time.

## Material and methods

### Subjects

KD patients who visited the West China Second Hospital of Sichuan University between April 2015 and October 2020 were prospectively recruited.

Inclusion criteria mandated patients to have a fever and to meet at least 4 out of the 5 diagnostic criteria for KD (rash, bilateral conjunctival injection, cervical lymph node involvement, mucous membrane changes, and limb changes), or at least 3 criteria along with documented coronary artery abnormalities on echocardiography, according to the American Heart Association guidelines (23). Structured questionnaires with pre-coded questions including basic demographic information, clinical manifestations, hematological examination results, treatment and follow-up outcomes, were used for data collection. All questionnaires were pretested and revised accordingly. Data collection was performed by two proficient physicians. The questionnaires were double-checked to assure its completeness. Informed written consent was obtained from parents after the nature of this study had been fully explained to them. The study was approved by the University Ethics Committee on Human Subjects at Sichuan University. All research was performed in accordance with relevant guidelines and regulations. During the COVID-19 pandemic, each patient underwent a pre-admission COVID-19 test, with all results indicating negativity prior to hospital admission.

The exclusion criteria encompassed patients with known congenital or chronic hematological disorders affecting the coagulation cascade, end-stage renal disease necessitating dialysis, acute or chronic hepatic failure, and autoimmune diseases. Additionally, individuals who had recently undergone surgery, patients with infectious or inflammatory conditions, and those receiving oral anticoagulants or heparin therapy were also excluded. Ultimately, a total of 523 patients were included in the study. Patients with Kawasaki Disease were categorized into two groups: those with Kawasaki Disease complicated by shock (KDSS group) and those without shock (non-KDSS group). In accordance with previously published guidelines, KDSS is defined as the presence of hypotension and shock (10). We diagnose KDSS if at least one of the following criteria is met: age-specific systolic hypotension (infants 0–28 days, <60 mmHg; infants aged 1–12 months, <70 mmHg; children aged 1–10 years, <70 mmHg + 2 times the age; children >10 years, ≤90 mmHg) (24, 25), a decrease in systolic blood pressure of ≥20% from baseline, or clinical symptoms of poor perfusion (tachycardia, prolonged capillary refill time, cold extremities, decreased pulse, oliguria, or altered mental status), regardless of blood pressure measurements (26).

The following data were collected for all patients: age, gender, typical clinical symptoms of KD, the presence of complete or incomplete KD, and IVIG resistance status. Echocardiographic data, including pericardial effusion, valvular regurgitation, cardiac enlargement, and coronary artery abnormalities, were also recorded. Laboratory parameters assessed in the results included white blood cell count, neutrophil-to-lymphocyte ratio (NLR), hemoglobin levels, platelet count, transaminases, albumin levels, total bilirubin, urea, creatinine, erythrocyte sedimentation rate (ESR), C-reactive protein (CRP), and coagulation function assessment. All laboratory parameters were collected prior to the onset of KDSS or IVIG therapy.

All patients received a continuous intravenous infusion of 2 g/kg of IVIG and 30–50 mg/kg/day of aspirin for a period of 24 h, which was continued until the cessation of fever. All KDSS patients received fluid resuscitation and vasoactive agents were given if the fluid resucitation did not work. Initial IVIG resistance was defined as the recurrence or persistence of fever or other clinical symptoms of KD for at least 36 h but not exceeding 7 days after the completion of the first intravenous immunoglobulin (IVIG) treatment. For patients exhibiting initial IVIG resistance, a second dose of IVIG (2 g/kg as a single intravenous infusion) was administered as per the expert consensus on the diagnosis and treatment of KD in China. Furthermore, in cases where patients continued to experience recurrent or persistent fever after the second IVIG administration, a pulse intravenous injection of methylprednisolone (10–30 mg/kg/day) was administered for a consecutive 3 days, followed by a gradual transition to oral prednisone (2 mg/kg/day) for a duration of 7 days.

As per the institutional protocol, standardized echocardiograms were obtained by experienced pediatric cardiologists during the acute/subacute phase and at the 6–8-week follow-up assessment in the cardiac outpatient clinic, continuing until coronary artery abnormalities were resolved. Coronary artery abnormalities were defined based on dimension *Z*-scores (normalized to body surface area) as follows: no involvement (*Z*-score <2.0), dilatation (*Z*-score ≥2.0 to <2.5), and aneurysm (*Z*-score ≥2.5), with giant aneurysms defined as a *Z*-score ≥10, depending on the maximum internal diameter of the right coronary artery, left anterior descending branch, and left circumflex artery.

### Statistics

Data analysis was performed with SPSS 27.0. (IBM SPSS Statistics version 27.0, Armonk, NY, IBM Corp.). Descriptive statistics for continuous variables were presented as medians (interquartile range, IQR) or means (standard deviations), while categorical variables were expressed as frequencies (%). Group differences in demographic characteristics, clinical presentations, and laboratory data were assessed using chi-square tests and unpaired Student's *t*-tests or Mann–Whitney *U*-tests as appropriate.

In the univariate analysis, key indicators that exhibited differences but lacked correlation with coagulation parameters were subjected to multivariable logistic regression analysis separately in order to identify independent predictive factors for the occurrence of KDSS. The predictive value of PT, d-dimer, FDP, and ATIII for KDSS occurrence was compared, and the optimal cutoff values and their predictive accuracy were determined through ROC analysis. Sensitivity, specificity, positive predictive value (PPV), negative predictive value (NPV), diagnostic accuracy, odds ratio (OR), area under the curve (AUC) of the Receiver Operating Characteristic (ROC) curve, and Youden's Index were evaluated. A significance level of *p* < .05 was considered statistically significant. The performance differences between different ROC curves were compared using the Delong test.

## Results

### The clinical features and serum coagulation profiles of patients with KDSS

As shown in Table 1, the KDSS group comprised 29 individuals, while the non-KDSS group included 494 individuals. For patients with KDSS, all patients received volume resuscitation, 11 cases (37.9%) received vasoactive infusions. There were no significant differences between the KDSS group and the non-KDSS group regarding gender, typical clinical presentations excluding cervical lymphadenopathy, and the incidence of incomplete Kawasaki disease. However, the KDSS group presented with older age and had a higher proportion with cervical lymphadenopathy. The incidences of pericardial effusion, valvular regurgitation, cardiac enlargement, CALs, and IVIG resisitance in the KDSS group were significantly higher than the non-KDSS group.Children in the KDSS group showed significant elevations in NLR, ALT, Total bilirubin, Creatinine, Urea nitrogen, and CRP, while Hemoglobin, PLT, AST, Sodium, and Potassium were significantly lower (*p* < .05).

**Table 1:** Comparison of clinical data between the KDSS groups and non-KDSS group.

	KDSS (*n* = 29)	Non-KDSS (*n* = 494)	*p*-value
Age (month)	51.1 [22.5–66.5]	32.8 [14.0–46.0]	.005*
Male (%)	15 (51.7)	281 (56.9)	.586
Clinical manifestations			
Rash, *n* (%)	25 (86.2)	371 (75.1)	.175
Bilateral bulbar conjunctive injection, *n* (%)	26 (89.7)	455 (92.1)	.637
Erythema of oral and pharyngeal mucosa, *n* (%)	26 (89.7)	455 (92.1)	.637
Edema and erythema of the extremities, *n* (%)	20 (69.0)	276 (55.9)	.167
Cervical lymphadenopathy, *n* (%)	24 (82.8)	202 (40.9)	<.001*
Incomplete KD, *n* (%)	7 (24.1)	147 (29.8)	.519
Pericardial effusion (%)	4 (13.8)	16 (3.2)	.004*
Valve regurgitation (%)	15 (51.7)	48 (9.7)	<.001*
Cardiac enlargement (%)	11 (37.9)	34 (6.9)	<.001*
Coronary artery lesions (CALs), *n* (%)	11 (37.9)	34 (6.9)	<.001*
IVIG resisitance, *n* (%)	17 (58.6)	70 (14.2)	<.001*
Laboratory features			
WBC count (10^9^/L)	15.71 [11.05–21.30]	14.12 [10.50–16.60]	.108
NLR	14.45 [7.20–15.53]	4.38 [1.83–5.28]	<.001^a,^*
Hemoglobin (g/L)	97.28 ± 15.90	108.86 ± 14.59	<.001*
PLT count (10^9^/L)	252.83 ± 125.22	348.94 ± 172.66	.003*
ALT (IU/L)	108.34 [36.00–152.50]	84.07 [19.00–88.00]	.002^a,^*
AST (IU/L)	68.52 [37.00–87.50]	69.23 [25.00–49.00]	<.001^a,^*
ALB (g/L)	30.19 ± 3.67	37.71 ± 5.14	<.001*
Total bilirubin (mg/L)	31.76 ± 39.06	9.25 ± 12.80	.004*
Creatinine (umol/L)	42.49 ± 31.29	27.90 ± 11.22	.018*
Urea nitrogen (mmol/L)	5.76 [2.98–6.90]	3.27 [2.20–3.43]	<.001^a,^*
Sodium (mmol/L)	133.37 [131.48–136.28]	136.24 [134.40–138.80]	<.001^a,^*
Potassium (mmol/L)	3.47 [3.20–3.80]	4.12 [3.73–4.50]	<.001^a,^*
ESR (mm/h)	62.26 ± 36.19	66.19 ± 27.71	.514
CRP (mg/L)	137.85 ± 51.32	81.75 ± 49.17	<.001*
PT (s)	14.98 [13.25–15.45]	13.62 [12.50–14.30]	.008*
APTT (s)	38.36 [31.45–43.75]	34.13 [29.00–37.03]	.012^a,^*
TT (s)	16.00 [15.00–16.55]	16.89 [15.70–16.80]	.011^a,^*
D-dimer (mg/L)	6.62 [2.07–7.89]	2.35 [0.81–2.28]	<.001^a,^*
Fibrinogen (mg/dl)	513.97 [393.50–638.50]	562.68 [470.00–662.00]	.074
FDP (ug/ml)	21.61 [8.15–26.90]	9.07 [3.88–9.70]	<.001^a,^*
ATIII (%)	68.97 [61.50–78.50]	89.54 [80.75–100.00]	<.001^a,^*

The data are presented as the median with the 25th and 75th percentiles in square brackets for continuous variables or mean (standard deviation) and as the percentage for the categorical variables.

WBC, white blood cell; NLR, neutrophil-to-lymphocyte ratio; PLT, platelet; ALT, alanine aminotransferase; AST, aspartate aminotransferase; ALB, albumin; ESR, erythrocyte sedimentation rate; CRP, C-reactive protein; PT, prothrombin time; APTT, activated partial thromboplastin time; TT, total thrombin time; FDP, fibrin degradation products; ATIII, antithrombin III; CALs, coronary artery lesions.

* Statistically significant (*p* < .05).

^a^ Variables between the two groups were compared by the Mann–Whitney *U*-test due to abnormal data distribution.

### Serum coagulation levels for predicting KDSS

The impact on the coagulation system was assessed by evaluating the coagulation profile of the KDSS group and the non-KDSS group. Children in the KDSS group had significantly longer PT (14.98 [13.25–15.45] s vs. 13.62 [12.50–14.30] s, *p* = .008), APTT (38.36 [31.45–43.75] s vs. 34.13 [29.00–37.03] s, *p* = .012), and shorter TT (16.00 [15.00–16.55] s vs. 16.89 [15.70–16.80] s, *p* = .011). D-dimer (6.62 [2.07–7.89] mg/L vs. 2.35 [0.81–2.28] mg/L, *p* < .001) and FDP (21.61 [8.15–26.90] ug/ml vs. 9.07 [3.88–9.70] ug/ml, *p* < .001) were significantly elevated in the KDSS group, while ATIII activity (68.97% [61.50%–78.50%] vs. 89.54% [80.75%–100.00%], *p* < .001) was significantly decreased. Fibrinogen (513.97 [393.50–638.50] mg/dl vs. 562.68 [470.00–662.00] mg/dl) showed no significant difference (Table 1).

### Multivariate logistic regression and ROC curve analysis were used to predict KDSS and validate the model's effectiveness

Pearson's correlation was applied to assess the correlation between individual factors and coagulation characteristics. Strong variables were excluded from the model (Supplementary Table S1). Multivariate logistic regression analysis indicated that longer PT, higher D-dimer and FDP levels, and lower ATIII activity were independent risk factors for the occurrence of KDSS, while APTT and TT did not reach significant differences in the multivariate logistic regression analysis (Table 2).

**Table 2:** A multivariate logistic regression model for KDSS group and non-KDSS group.

Variates	*β*	SE	Walds	*p*-value	OR	95% CI
Age	0.015	0.007	4.081	0.043*	1.015	1.000–1.030
Cervical lymphadenopathy	2.033	0.591	11.845	0.001*	7.634	2.399–24.293
ALT	0.001	0.002	0.328	0.567	1.001	0.997–1.005
AST	−0.001	0.003	0.096	0.757	0.999	0.994–1.004
Urea nitrogen	0.016	0.014	1.208	0.272	1.016	0.988–1.045
ATIII	−0.066	0.012	31.175	<0.001*	0.936	0.915–0.958
Age	0.002	0.008	0.045	0.832	1.002	0.987–1.017
Cervical lymphadenopathy	1.700	0.557	9.317	0.002*	5.472	1.837–16.296
PLT	−0.008	0.003	9.935	0.002*	0.992	0.987–0.997
ALT	0.002	0.002	0.775	0.379	1.002	0.998–1.007
AST	−0.002	0.003	0.337	0.562	0.998	0.992–1.004
Creatinine	0.031	0.010	10.171	0.001*	1.032	1.012–1.052
Urea nitrogen	0.013	0.018	0.525	0.469	1.013	0.979–1.048
PT	0.137	0.043	10.400	0.001*	1.147	1.055–1.247
Age	−0.005	0.009	0.332	0.564	0.995	0.978–1.012
Cervical lymphadenopathy	1.433	0.586	5.970	0.015	4.190	1.328–13.221
PLT	−0.005	0.003	3.317	0.069	0.995	0.990–1.000
ALT	−0.001	0.003	0.030	0.863	0.999	0.994–1.005
AST	0.000	0.003	0.003	0.955	1.000	0.995–1.005
Total bilirubin	0.022	0.009	5.519	0.019	1.022	1.004–1.041
Creatinine	0.031	0.011	8.147	0.004	1.031	1.010–1.054
Urea nitrogen	0.012	0.018	0.463	0.496	1.012	0.977–1.049
Sodium	−0.009	0.020	0.213	0.644	0.991	0.953–1.030
Potassium	−1.386	0.424	10.687	0.001	0.250	0.109–0.574
APTT	0.011	0.010	1.132	0.287	1.011	0.991–1.031
Cervical lymphadenopathy	1.630	0.690	5.575	0.018	5.105	1.319–19.757
NLR	0.020	0.035	0.347	0.556	1.021	0.954–1.092
Hemoglobin	−0.065	0.023	7.710	0.005	0.937	0.895–0.981
PLT	−0.003	0.003	1.365	0.243	0.997	0.992–1.002
ALT	0.001	0.003	0.075	0.784	1.001	0.995–1.007
AST	0.000	0.002	0.000	0.996	1.000	0.996–1.004
Total bilirubin	0.019	0.012	2.544	0.111	1.019	0.996–1.042
ALB	−0.143	0.043	10.984	0.001	0.867	0.797–0.943
Creatinine	0.027	0.012	4.977	0.026	1.028	1.003–1.052
Urea nitrogen	0.018	0.021	0.739	0.390	1.019	0.977–1.062
Sodium	−0.004	0.025	0.024	0.876	0.996	0.949–1.046
Potassium	−0.664	0.507	1.718	0.190	0.515	0.191–1.389
CRP	0.004	0.006	0.387	0.534	1.004	0.992–1.015
TT	−0.198	0.249	0.629	0.428	0.821	0.504–1.338
Age	0.006	0.009	0.525	.469	1.006	0.989–1.024
Cervical lymphadenopathy	1.497	0.602	6.188	.013*	4.470	1.374–14.541
Hemoglobin	−0.075	0.021	12.966	<.001*	0.928	0.891–0.967
PLT	−0.003	0.002	1.833	.176	0.997	0.992–1.002
ALT	−0.001	0.003	0.103	.748	0.999	0.993–1.005
AST	0.000	0.003	0.024	.877	1.000	0.995–1.006
Total bilirubin	0.022	0.010	4.517	.034*	1.023	1.002–1.044
Urea nitrogen	0.017	0.015	1.288	.256	1.017	0.988–1.047
Sodium	0.001	0.019	0.006	.940	1.001	0.965–1.040
Potassium	−1.126	0.433	6.752	.009*	0.324	0.139–0.758
D-dimer	0.092	0.037	6.262	.012*	1.096	1.020–1.177
Hemoglobin	−0.057	0.021	7.396	0.007*	0.944	0.906–0.984
PLT count	−0.005	0.002	3.839	0.050	0.995	0.991–1.000
ALT	0.002	0.003	0.680	0.409	1.002	0.997–1.008
AST	−0.001	0.002	0.159	0.690	0.999	0.995–1.003
Total bilirubin	0.029	0.010	8.580	0.003*	1.030	1.010–1.050
ALB	−0.133	0.038	12.347	0.000*	0.875	0.812–0.943
Creatinine	0.027	0.010	6.927	0.008*	1.027	1.007–1.048
Urea nitrogen	0.026	0.020	1.768	0.184	1.026	0.988–1.067
Sodium	−0.001	0.020	0.005	0.941	0.999	0.961–1.038
Potassium	−0.874	0.430	4.143	0.042*	0.417	0.180–0.968
Fibrinogen	0.000	0.002	0.060	0.807	1.000	0.996–1.003
Age	−0.005	0.008	0.375	0.540	0.995	0.978–1.011
Cervical lymphadenopathy	1.592	0.574	7.697	0.006*	4.914	1.596–15.130
PLT	−0.004	0.003	2.079	0.149	0.996	0.991–1.001
ALT	0.000	0.003	0.003	0.956	1.000	0.994–1.006
AST	0.000	0.002	0.009	0.925	1.000	0.996–1.005
Creatinine	0.035	0.011	10.423	0.001*	1.035	1.014–1.057
Urea nitrogen	0.012	0.018	0.487	0.485	1.012	0.978–1.048
Sodium	−0.007	0.020	0.146	0.702	0.993	0.955–1.031
Potassium	−1.533	0.411	13.877	<.001*	0.216	0.096–0.484
FDP	0.020	0.010	3.978	0.046*	1.020	1.000–1.040

ALT, alanine aminotransferase; AST, Aspartate aminotransferase; ATIII, antithrombin III; PLT, platelet; PT, prothrombin time; APTT, activated partial thromboplastin time; NLR, neutrophil-to-lymphocyte ratio; ALB, albumin; CRP, C-reactive protein; TT, total thrombin time; FDP, fibrin degradation products.

* Statistically significant (*p* < .05).

**Figure 1:**
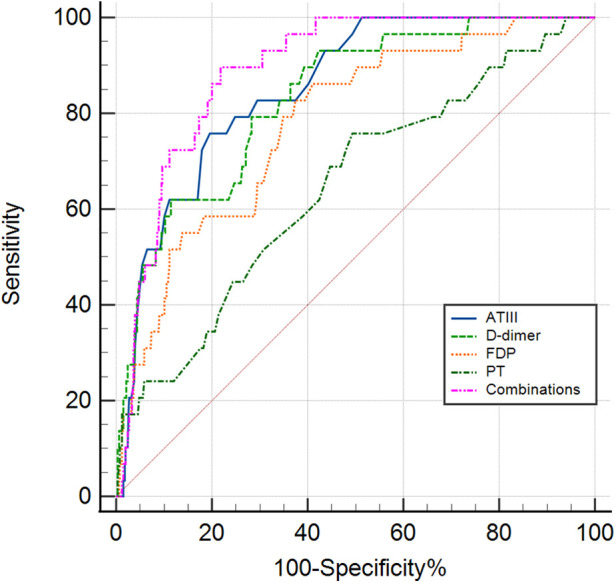
The receiver operating characteristic (ROC) curve for coagulation profiles in predicting the KDSS group and non-KDSS group. ATIII, antithrombin III; FDP, fibrin degradation products; PT, prothrombin time; Combinations, the combinations of the four indicators of ATIII, D-dimer, FDP and PT.

ROC curve analysis was conducted to assess the serum coagulation profile's predictive ability for KDSS occurrence. The areas under the curves (AUC) for PT, D-dimer, FDP, and ATIII in predicting KDSS occurrence were 0.646, 0.831, 0.779, and 0.852, respectively. The cutoff values for PT, D-dimer, FDP, and ATIII were 13.45 s, 2.03 mg/L, 7.45 ug/ml, and 77.5%, respectively, with sensitivities of 76%, 79%, 83%, and 76%, and specificities of 51%, 72%, 63%, and 80%, respectively (Table 3 and Figure 1). When we performed a combined ROC curve analysis of the four indicators, the AUC was 0.891, with a sensitivity of 0.90 and a specificity of 0.78. The Delong test showed that the combined analysis of the four indicators was significantly better than the analysis of each indicator individually (Table 3 and Supplementary Data Sheet S1).

**Table 3:** The validity of coagulation profiles in predicting the KDSS group and non-KDSS group.

KDSS	Diagnostic test	Gold standard			Sen	Spe	PPV	NPV	Diagnostic accuracy	OR (95% CI)	*p*	AUC	Youden's index
Total group (*n* = 523)	PT ≥13.45 s	Positive	22	244	0.76	0.51	0.08	0.97	0.52	3.17 (1.33–7.55)	.006*	0.646	0.265
		Negative	7	250									
	D-dimer ≥2.03 mg/L	Positive	23	139	0.79	0.72	0.14	0.98	0.72	9.79 (3.90–24.56)	<.001*	0.831	0.510
		Negative	6	355									
	FDP ≥7.45 ug/ml	Positive	24	184	0.83	0.63	0.12	0.98	0.64	8.09 (3.03–21.56)	<.001*	0.779	0.454
		Negative	5	310									
	ATIII ≤77.5%	Positive	22	97	0.76	0.80	0.19	0.98	0.80	12.86 (5.34–30.98)	<.001*	0.852	0.563
		Negative	7	397									
	Combinations	Positive	26	109	0.90	0.78	0.19	0.99	0.79	30.61 (9.09–103.05)	<.001*	0.891	0.678
		Negative	3	386									

PT, prothrombin time; FDP, fibrin degradation products; ATIII, antithrombin III; Sen, sensitivity; Spe, specificity; PPV, positive predictive value; NPV, negative predictive value; OR, odds ratio; CI, confidence ratio; AUC, area under the curve.

Combinations: The combinations of the four indicators of ATIII, D-dimer, FDP, and PT.

* Statistically significant (*p* < .05).

## Discussion

In this study, we observed a higher incidence of adverse events, including IVIG resistance and cardiac complication (pericardial effusion, valvular regurgitation, cardiac enlargement, coronary artery damage), in patients belonging to the KDSS group. These Patients exhibited significantly extensive inflammatory burden. Notably, our study is the first to comprehensively describe coagulation profiles in KDSS patients and further support the hypothesis of significant differences in their coagulation profile: longer PT and APTT durations, elevated D-dimer and FDP levels, as well as reduced fibrinogen levels and ATIII activity. Additionally, through multivariate logistic regression analysis, we identified longer PT duration, higher D-dimer levels, and lower ATIII activity as independent risk factors for developing KDSS. The ROC curve analysis results showed that D-dimer and ATIII activity had good predictive value for the occurrence of KDSS, and the combined analysis of the four indicators had the best predictive performance. Our study results suggest that patients with impaired coagulation function may require more aggressive treatment to reduce the likelihood of developing KDSS.

Currently, the pathogenesis of KDSS remains elusive; however, it is speculated to involve multiple mechanisms, including severe systemic vasculitis-induced capillary leakage, immune dysregulation, systemic inflammatory responses due to infections, myocardial dysfunction, and abnormal cytokine regulation (27, 28). In a study conducted by Yandie Li et al., significantly higher levels of cytokines such as IL-6, IL-10, TNF-α, and IFN-γ were observed in the serum of KDSS patients compared to the control group consisting of KD patients (14). Our study revealsed that the KDSS group exhibited significantly elevated levels of NLR and CRP along with a more severe degree of anemia. These findings have been closely associated with increased cytokine levels. For instance, the pronounced anemia observed in KDSS patients may result from the upregulation of hepcidin induced by IL-6 (29). CRP levels are positively correlated with IL-6 (30). Elevated IL-6 levels promote chronic inflammation, leading to multi-organ damage and failure. With sustained elevation of IL-6, patients may experience hypoalbuminemia, hyponatremia, and anemia (31). Our study results indicate that KDSS patients in the study group exhibited significantly lower levels of blood sodium, blood potassium, and albumin compared to the KD group, reflecting the substantial elevation of cytokines, including IL-6, in KDSS patients. Furthermore, higher levels of proinflammatory cytokines, including TNF-α, IL-6, and IL-8, were found in patients with elevated D-dimer levels. TNF-α is one of the key cytokines in KD and can induce vascular endothelial cell damage through oxidative stress, inflammation, and apoptosis (19). The vascular endothelium is covered with a network of glycosaminoglycans and glycoproteins (glycocalyx), and if the glycocalyx is damaged due to KD vasculitis, endothelial anticoagulant properties are compromised, leading to platelet aggregation abnormalities, a procoagulant state, and increased fibrinolysis (32). In consist with above findings, the present study clinically verified our primary hypothesis that patients with KDSS had significantly decreased PLT levels and elevated D-dimer levels.The condition in children and adolescents associated with COVID-19, known as Multisystem Inflammatory Syndrome in Children (MIS-C), is characterized by acute systemic inflammation and multi-organ dysfunction. A significant proportion of MIS-C patients exhibit similar manifestations to those observed in KDSS, with many MIS-C cases presenting signs of coagulation abnormalities (prolonged PT or APTT, elevated D-dimer) (33).

In our study, patients in the KDSS group also displayed marked coagulation dysfunction, suggesting that the occurrence of coagulation abnormalities is not incidental and may be associated with the body's excessive inflammatory response. Therefore, coagulation dysfunction can serve as an effective indicator for predicting the development of KDSS. Moreover, prolonged PT and APTT may be attributed to reduced synthesis and/or increased consumption of coagulation factors (34). In clinical practice, it is well known that KD patients often exhibit moderate liver damage accompanied by hepatomegaly. Activation of NK cells by bacterial superantigens can lead to their accumulation in endothelial cells and hepatic sinusoidal endothelial cells, contributing to vascular endothelial cell damage and hepatocellular injury associated with KD (35). The significant increase in neutrophils in KD patients results in the substantial production of neutrophil extracellular traps (NETs), which act as procoagulants, creating a prothrombotic environment. Additionally, NETs can stimulate the activation of the complement pathway. Neutrophils, NETs, complement, and thrombus formation all play roles in liver damage (36). The liver, being involved in maintaining coagulation function, contributes to the synthesis and metabolism of clotting factors such as PT and APTT. Therefore, when KD patients experience concomitant liver dysfunction, it can impact coagulation function since most clotting factors are synthesized in the liver, affecting their production. The association between liver damage and alterations in coagulation function highlights the intricate interplay between immune responses, liver function, and coagulation in KD patients. In fact, in our study, patients in the KDSS group had significantly elevated ALT and total bilirubin, hinting liver dysfunction. Overall, the prolongation of PT and APTT may also be partially attributed to more severe liver dysfunction in KDSS patients, reflecting the severe inflammatory reaction.

Due to the higher incidence of IVIG resistance and cardiovascular complications such as CALs in KDSS patients, early identification of KDSS is crucial to mitigate adverse outcomes. However, there is currently limited research on predicting KDSS, with most relevant studies primarily summarizing the epidemiology and certain laboratory or imaging differences in KDSS patients (5–7, 9, 10, 28, 37–42). A case-control study encompassing 27 KDSS patients and 43 KD patients as controls revealed distinct characteristics of KDSS, including heightened cytokine production, a higher likelihood of IVIG unresponsiveness, and an increased incidence of coronary artery abnormalities. These findings align with our study results. The ROC curve analysis demonstrated that the sensitivity and specificity of IL-6 at 66.7 pg/ml, IL-10 at 20.85 pg/ml, and IFN-γ at 8.35 pg/ml for predicting KDSS were 85.2% and 62.8%, 66.7% and 83.7%, and 74.1% and 74.4%, respectively (13). However, these predictive indicators either exhibited lower sensitivity or lower specificity. In another retrospective analysis involving 13 KDSS patients, 35 patients with infectious shock, and 91 control KD patients, ROC curve indicated that the optimal ESR cutoff value for diagnosing KDSS was 56.0 mm/h (sensitivity 75.0%, specificity 100.0%), and the optimal creatinine cutoff value for diagnosing TSS was 0.695 (sensitivity 76.9%, specificity 84.6%). Historically, there has been a lack of relevant research on predicting KDSS occurrence through coagulation profiles. Our study represents the first comprehensive depiction of the coagulation function in KDSS patients, revealing that factors such as antithrombin III (ATIII) can be utilized for predicting KDSS development, exhibiting high predictive efficacy.

The strengths of this study lie in its demonstration of the predictive value of the coagulation profile for KDSS occurrence, along with a relatively large sample size. However, the study has some limitations. First, it was conducted at a single institution, which may introduce selection bias as the hospital is a major pediatric medical center in the southwestern region of China, possibly leading to a higher number of critically ill patients. Despite these limitations, our study is the first to reveal the predictive value of the coagulation profile for the occurrence of KDSS and provides evidence of significant differences in PT, APTT, TT, D-dimer, FDP levels, and ATIII activity between the KDSS group and non-KDSS group. These changes may serve as supplementary laboratory biomarkers for predicting KDSS occurrence. Furthermore, D-dimer and ATIII activity as a single biomarker for predicting KDSS occurrence may have relatively high sensitivity and specificity.

## Conclusion

In summary, KDSS is characterized by older age, a higher likelihood of pericardial effusion, valvular regurgitation, cardiac enlargement, coronary artery damage, and IVIG resistance. There is an increased risk of KDSS occurrence in older children with KD who exhibit a procoagulant state during the acute phase.

## Data Availability

The original contributions presented in the study are included in the article/Supplementary Material, further inquiries can be directed to the corresponding authors.
