# Supplementary material for: Predictive value of coagulation profiles for Kawasaki disease shock syndrome: a prospective cohort study
Source: Front Pediatr. 2024 Aug 16;12:1450710. doi: 10.3389/fped.2024.1450710 (PMC11362036; doi:10.3389/fped.2024.1450710)
Supplement: Supplementary file 1 [file Table1.pdf]

Supplementary Table 1: The correlation analysis of Clinical and laboratory indicators

		Age (mo)	Cervical lymphadenopathy	Incomplete KD	NLR	Hemoglobin	PLT count	AST	ALT	ALB	Total bilirubin	Creatinine	Urea nitrogen	Sodium	Potassium	CRP	PT	APTT	TT	D-dimer	FDP	ATIII
Age (mo)	Pearson correlation	1	.311**	-0.010	.468**	.168**	-.169**	0.016	0.085	-.087*	.161**	.365**	.131**	-0.006	-.248**	.130**	-0.036	-0.058	.102*	-0.007	-0.040	0.054
	Significance (double tail)		0.000	0.821	0.000	0.000	0.000	0.709	0.053	0.046	0.000	0.000	0.003	0.884	0.000	0.003	0.406	0.181	0.020	0.872	0.359	0.217
	number of cases	524	524	524	524	524	523	521	521	521	521	517	516	516	517	521	524	524	524	524	524	524
Cervical lymphadenopathy	Pearson correlation	.311**	1	.178**	.311**	.100*	-.098*	-0.003	0.079	-0.015	.208**	.150**	0.077	-0.028	-.087*	.105*	0.048	-0.009	0.060	-0.010	0.045	0.004
	Significance (double tail)	0.000		0.000	0.000	0.022	0.025	0.953	0.073	0.734	0.000	0.001	0.081	0.531	0.047	0.016	0.268	0.834	0.173	0.826	0.304	0.927
	number of cases	524	524	524	524	524	523	521	521	521	521	517	516	516	517	521	524	524	524	524	524	524
Incomplete KD	Pearson correlation	-0.010	.178**	1	0.001	.086*	-0.065	0.044	0.055	0.051	-0.081	0.003	0.020	-0.064	-0.048	-0.039	-0.084	-0.041	0.026	0.012	-0.052	0.031
	Significance (double tail)	0.821	0.000		0.976	0.049	0.135	0.322	0.208	0.244	0.066	0.950	0.657	0.145	0.278	0.378	0.055	0.349	0.547	0.785	0.232	0.473
	number of cases	524	524	524	524	524	523	521	521	521	521	517	516	516	517	521	524	524	524	524	524	524
NLR	Pearson correlation	.468**	.311**	0.001	1	-0.056	-.152**	0.023	.141**	-.295**	.445**	.432**	.139**	-.162**	-.374**	.359**	.115**	.093*	0.038	.100*	.144**	-.213**
	Significance (double tail)	0.000	0.000	0.976		0.200	0.000	0.596	0.001	0.000	0.000	0.000	0.001	0.000	0.000	0.000	0.008	0.034	0.383	0.022	0.001	0.000

	number of cases	524	524	524	524	524	523	521	521	521	521	517	516	516	517	521	524	524	524	524	524	524
Hemoglobin	Pearson correlation	.168**	.100*	.086*	-0.056	1	0.038	0.052	.089*	.418**	-0.007	0.065	0.025	.124**	.153**	-.249**	-.143**	-.136**	0.001	-0.018	-.111*	.212**
	Significance (double tail)	0.000	0.022	0.049	0.200		0.391	0.240	0.041	0.000	0.865	0.143	0.573	0.005	0.000	0.000	0.001	0.002	0.981	0.685	0.011	0.000
	number of cases	524	524	524	524	524	523	521	521	521	521	517	516	516	517	521	524	524	524	524	524	524
PLT count	Pearson correlation	-.169**	-.098*	-0.065	-.152**	0.038	1	-0.068	-.097*	.157**	-.112*	-.162**	-0.017	0.044	.147**	-.205**	-0.064	-0.079	-0.004	-0.045	-0.052	.145**
	Significance (double tail)	0.000	0.025	0.135	0.000	0.391		0.124	0.027	0.000	0.010	0.000	0.701	0.321	0.001	0.000	0.143	0.072	0.923	0.302	0.231	0.001
	number of cases	523	523	523	523	523	523	521	521	521	521	517	516	516	517	520	523	523	523	523	523	523
AST	Pearson correlation	0.016	-0.003	0.044	0.023	0.052	-0.068	1	.791**	0.045	.105*	0.031	0.008	0.070	.097*	0.047	-0.013	-0.022	0.005	0.006	0.027	0.000
	Significance (double tail)	0.709	0.953	0.322	0.596	0.240	0.124		0.000	0.310	0.016	0.479	0.863	0.111	0.027	0.287	0.771	0.619	0.902	0.888	0.540	0.993
	number of cases	521	521	521	521	521	521	521	521	521	521	517	516	515	516	518	521	521	521	521	521	521
ALT	Pearson correlation	0.085	0.079	0.055	.141**	.089*	-.097*	.791**	1	0.018	.270**	0.064	-0.003	0.066	0.001	.094*	0.053	0.029	-0.026	0.004	0.069	-0.058
	Significance (double tail)	0.053	0.073	0.208	0.001	0.041	0.027	0.000		0.679	0.000	0.144	0.948	0.135	0.977	0.033	0.231	0.514	0.561	0.923	0.116	0.186
	number of cases	521	521	521	521	521	521	521	521	521	521	517	516	515	516	518	521	521	521	521	521	521

ALB	Pearson correlation	-.087*	-0.015	0.051	-.295**	.418**	.157**	0.045	0.018	1	-.116**	-.112*	0.025	.148**	.390**	-.408**	-.175**	-.177**	0.046	-.160**	-.152**	.449**
	Significance (double tail)	0.046	0.734	0.244	0.000	0.000	0.000	0.310	0.679		0.008	0.011	0.571	0.001	0.000	0.000	0.000	0.000	0.297	0.000	0.001	0.000
	number of cases	521	521	521	521	521	521	521	521	521	521	517	516	515	516	518	521	521	521	521	521	521
Total bilirubin	Pearson correlation	.161**	.208**	-0.081	.445**	-0.007	-.112*	.105*	.270**	-.116**	1	.224**	0.062	-0.037	-.203**	.180**	.127**	0.056	-0.027	0.046	.091*	-.168**
	Significance (double tail)	0.000	0.000	0.066	0.000	0.865	0.010	0.016	0.000	0.008		0.000	0.157	0.401	0.000	0.000	0.004	0.200	0.538	0.300	0.037	0.000
	number of cases	521	521	521	521	521	521	521	521	521	521	517	516	515	516	518	521	521	521	521	521	521
Creatinine	Pearson correlation	.365**	.150**	0.003	.432**	0.065	-.162**	0.031	0.064	-.112*	.224**	1	.185**	-0.055	-.177**	.111*	0.036	0.010	0.000	.097*	0.021	-.096*
	Significance (double tail)	0.000	0.001	0.950	0.000	0.143	0.000	0.479	0.144	0.011	0.000		0.000	0.211	0.000	0.011	0.418	0.828	0.999	0.027	0.632	0.029
	number of cases	517	517	517	517	517	517	517	517	517	517	517	516	513	514	515	517	517	517	517	517	517
Urea nitrogen	Pearson correlation	.131**	0.077	0.020	.139**	0.025	-0.017	0.008	-0.003	0.025	0.062	.185**	1	-0.022	-0.024	0.047	-0.031	-0.020	0.011	0.021	-0.005	0.007
	Significance (double tail)	0.003	0.081	0.657	0.001	0.573	0.701	0.863	0.948	0.571	0.157	0.000		0.619	0.588	0.291	0.483	0.643	0.808	0.642	0.916	0.868
	number of cases	516	516	516	516	516	516	516	516	516	516	516	516	512	513	514	516	516	516	516	516	516
	Pearson correlation	-0.006	-0.028	-0.064	-.162**	.124**	0.044	0.070	0.066	.148**	-0.037	-0.055	-0.022	1	.256**	-.146**	-.103*	-0.052	0.010	-0.036	-0.028	.147**

	Significance (double tail)	0.884	0.531	0.145	0.000	0.005	0.321	0.111	0.135	0.001	0.401	0.211	0.619		0.000	0.001	0.020	0.240	0.829	0.410	0.527	0.001
	number of cases	516	516	516	516	516	516	515	515	515	515	513	512	516	516	513	516	516	516	516	516	516
Potassium	Pearson correlation	-.248**	-.087*	-0.048	-.374**	.153**	.147**	.097*	0.001	.390**	-.203**	-.177**	-0.024	.256**	1	-.253**	-.202**	-0.080	0.070	-0.076	-0.046	.257**
	Significance (double tail)	0.000	0.047	0.278	0.000	0.000	0.001	0.027	0.977	0.000	0.000	0.000	0.588	0.000		0.000	0.000	0.068	0.113	0.086	0.293	0.000
	number of cases	517	517	517	517	517	517	516	516	516	516	514	513	516	517	514	517	517	517	517	517	517
CRP	Pearson correlation	.130**	.105*	-0.039	.359**	-.249**	-.205**	0.047	.094*	-.408**	.180**	.111*	0.047	-.146**	-.253**	1	.173**	.178**	0.012	.141**	.150**	-.398**
	Significance (double tail)	0.003	0.016	0.378	0.000	0.000	0.000	0.287	0.033	0.000	0.000	0.011	0.291	0.001	0.000		0.000	0.000	0.783	0.001	0.001	0.000
	number of cases	521	521	521	521	521	520	518	518	518	518	515	514	513	514	521	521	521	521	521	521	521
PT	Pearson correlation	-0.036	0.048	-0.084	.115**	-.143**	-0.064	-0.013	0.053	-.175**	.127**	0.036	-0.031	-.103*	-.202**	.173**	1	.336**	-0.050	0.002	0.011	-.248**
	Significance (double tail)	0.406	0.268	0.055	0.008	0.001	0.143	0.771	0.231	0.000	0.004	0.418	0.483	0.020	0.000	0.000		0.000	0.253	0.959	0.810	0.000
	number of cases	524	524	524	524	524	523	521	521	521	521	517	516	516	517	521	524	524	524	524	524	524
APTT	Pearson correlation	-0.058	-0.009	-0.041	.093*	-.136**	-0.079	-0.022	0.029	-.177**	0.056	0.010	-0.020	-0.052	-0.080	.178**	.336**	1	-0.061	0.005	-0.024	-.173**

	Significance (double tail)	0.181	0.834	0.349	0.034	0.002	0.072	0.619	0.514	0.000	0.200	0.828	0.643	0.240	0.068	0.000	0.000		0.161	0.910	0.584	0.000
	number of cases	524	524	524	524	524	523	521	521	521	521	517	516	516	517	521	524	524	524	524	524	524
TT	Pearson correlation	.102*	0.060	0.026	0.038	0.001	-0.004	0.005	-0.026	0.046	-0.027	0.000	0.011	0.010	0.070	0.012	-0.050	-0.061	1	-0.012	-0.046	0.034
	Significance (double tail)	0.020	0.173	0.547	0.383	0.981	0.923	0.902	0.561	0.297	0.538	0.999	0.808	0.829	0.113	0.783	0.253	0.161		0.790	0.289	0.436
	number of cases	524	524	524	524	524	523	521	521	521	521	517	516	516	517	521	524	524	524	524	524	524
D-dimer	Pearson correlation	-0.007	-0.010	0.012	.100*	-0.018	-0.045	0.006	0.004	-.160**	0.046	.097*	0.021	-0.036	-0.076	.141**	0.002	0.005	-0.012	1	.284**	-.115**
	Significance (double tail)	0.872	0.826	0.785	0.022	0.685	0.302	0.888	0.923	0.000	0.300	0.027	0.642	0.410	0.086	0.001	0.959	0.910	0.790		0.000	0.008
	number of cases	524	524	524	524	524	523	521	521	521	521	517	516	516	517	521	524	524	524	524	524	524
FDP	Pearson correlation	-0.040	0.045	-0.052	.144**	-.111*	-0.052	0.027	0.069	-.152**	.091*	0.021	-0.005	-0.028	-0.046	.150**	0.011	-0.024	-0.046	.284**	1	-.130**
	Significance (double tail)	0.359	0.304	0.232	0.001	0.011	0.231	0.540	0.116	0.001	0.037	0.632	0.916	0.527	0.293	0.001	0.810	0.584	0.289	0.000		0.003
	number of cases	524	524	524	524	524	523	521	521	521	521	517	516	516	517	521	524	524	524	524	524	524
ATIII	Pearson correlation	0.054	0.004	0.031	-.213**	.212**	.145**	0.000	-0.058	.449**	-.168**	-.096*	0.007	.147**	.257**	-.398**	-.248**	-.173**	0.034	-.115**	-.130**	1
	Significance (double tail)	0.217	0.927	0.473	0.000	0.000	0.001	0.993	0.186	0.000	0.000	0.029	0.868	0.001	0.000	0.000	0.000	0.000	0.436	0.008	0.003	

	number of cases	524	524	524	524	524	523	521	521	521	521	517	516	516	517	521	524	524	524	524	524	524

Abbreviations: NLR, neutrophil-to-lymphocyte ratio; PLT, platelet; ALT, alanine aminotransferase; AST, aspartate aminotransferase; ALB, albumin; CRP, C-reactive protein; PT, prothrombin time; APTT, activated partial thromboplastin time; TT, total thrombin time; FDP, fibrin degradation products; ATIII, antithrombin III.

*Statistically significant ($P < .05$)
