# Supplementary material for: Predictive value of coagulation profiles for Kawasaki disease shock syndrome: a prospective cohort study
Source: Front Pediatr. 2024 Aug 16;12:1450710. doi: 10.3389/fped.2024.1450710 (PMC11362036; doi:10.3389/fped.2024.1450710)
Supplement: Supplementary file 2 [file Datasheet1.pdf]

Comparison of ROC curves

Variable 1	ATIII
Variable 2	FDP
Variable 3	D_dimer<br>D-dimer
Variable 4	PT
Variable 5	Combination
Classification variable	KDSS

Sample size	523
Positive group [a]	29 (5.54%)
Negative group [b]	494 (94.46%)

[a] KDSS = 1
[b] KDSS = 0

Variable	AUC	SE [a]	95% CI [b]
ATIII	0.852	0.0304	0.819 to 0.881
FDP	0.779	0.0424	0.741 to 0.814
D_dimer	0.831	0.0364	0.796 to 0.862
PT	0.646	0.0549	0.603 to 0.687
Combination	0.891	0.0218	0.861 to 0.916

[a] DeLong et al., 1988
[b] Binomial exact

Pairwise comparison of ROC curves

ATIII ~ FDP	
Difference between areas	0.0728
Standard Error [a]	0.0583
95% Confidence Interval	-0.0415 to 0.187
z statistic	1.248
Significance level	P = 0.2119
**ATIII ~ D_dimer**	
Difference between areas	0.0209
Standard Error [a]	0.0526
95% Confidence Interval	-0.0822 to 0.124
z statistic	0.398
Significance level	P = 0.6906
**ATIII ~ PT**	
Difference between areas	0.206
Standard Error [a]	0.0586
95% Confidence Interval	0.0911 to 0.321
z statistic	3.514
Significance level	P = 0.0004
**ATIII ~ Combination**	
Difference between areas	0.0390
Standard Error [a]	0.0182
95% Confidence Interval	0.00336 to 0.0747
z statistic	2.144
Significance level	P = 0.0320
**FDP ~ D_dimer**	

Difference between areas	0.0518
Standard Error [a]	0.0206
95% Confidence Interval	0.0115 to 0.0922
z statistic	2.518
Significance level	P = 0.0118
FDP ~ PT	
Difference between areas	0.133
Standard Error [a]	0.0702
95% Confidence Interval	-0.00452 to 0.271
z statistic	1.896
Significance level	P = 0.0580
FDP ~ Combination	
Difference between areas	0.112
Standard Error [a]	0.0465
95% Confidence Interval	0.0207 to 0.203
z statistic	2.406
Significance level	P = 0.0161
D_dimer ~ PT	
Difference between areas	0.185
Standard Error [a]	0.0694
95% Confidence Interval	0.0489 to 0.321
z statistic	2.664
Significance level	P = 0.0077
D_dimer ~ Combination	
Difference between areas	0.0600
Standard Error [a]	0.0411
95% Confidence Interval	-0.0206 to 0.141
z statistic	1.459
Significance level	P = 0.1445
PT ~ Combination	
Difference between areas	0.245
Standard Error [a]	0.0586
95% Confidence Interval	0.130 to 0.360
z statistic	4.181
Significance level	P < 0.0001

[a] DeLong et al., 1988
